# Numerical Response of Owls to the Dampening of Small Mammal Population Cycles in Latvia

**DOI:** 10.3390/life13020572

**Published:** 2023-02-17

**Authors:** Andris Avotins, Andris Avotins, Viesturs Ķerus, Ainars Aunins

**Affiliations:** 1 Department of Zoology and Animal Ecology, Faculty of Biology, University of Latvia, Jelgavas Iela 1, LV-1004 Riga, Latvia; 2 Latvian Ornithological Society, Skolas Iela 3, LV-1010 Riga, Latvia

**Keywords:** diet, breeding performance, population trends, *Aegolius funereus*, *Asio otus*, *Strix aluco*, *Strix uralensis*, *Bubo bubo*

## Abstract

**Simple Summary:**

This article demonstrates the dampening of small mammal population dynamics and describes the numerical response of owls in Latvia. Numerical response was measured by diet, breeding performance, and trends in population change of six owl species. The responses varied among owl species, ranging from increased food niche breadth in more plastic species to reduced breeding performance and decreasing population size in more specialized species. The eagle owl seems to depend on voles in the previous autumn via the carry-over effect as measured by reduced breeding performance. Species more specialized in breeding in mature forests showed greater population declines, since mature forests are vital for owl breeding, as well as hold higher vole densities.

**Abstract:**

Strong numerical and functional responses of owls to voles in cyclic environments are well known. However, there is insufficient knowledge from the boreonemoral region in particular, with depleted populations of small mammals. In this study, we describe the dynamics of the small mammal population in Latvia from 1991 to 2016 and link them to owl population characteristics. We used food niche breadth, number of fledglings, and population trends to lay out the numerical response of six owl species to dampened small mammal population cycles. We found temporarily increasing food niche breadth in tawny and Ural owls. There were no other responses in the tawny owl, whereas the breeding performance of three forest specialist species—pygmy, Tengmalm’s, and Ural owls—corresponded to the vole crash years in Fennoscandia. Moreover, the populations of forest specialist owls decreased, and the change in the Ural owl population can be attributed to the depletion of small mammal populations. We found evidence of a carry-over effect in the eagle owl arising from a strong correlation of declining breeding performance with the small mammal abundance indices in the previous autumn. We conclude that dampening of the small mammal population cycles is an important covariate of the likely effects of habitat destruction that needs to be investigated further, with stronger responses in more specialized (to prey or habitat) species.

## 1. Introduction

Small mammals play an important role in various ecological processes. This role ranges from influence on a natural succession [1] through influence on plant and microorganism community composition and chemistry [2] to demographic processes of small mammal predators [3,4,5,6] and even population processes and the behavior of directly unrelated species [7,8,9]. Fluctuations in the availability of small mammals are known to affect the demography of several species of birds of prey, and the analysis of the breeding performance of the latter can reveal large-scale spatiotemporal patterns of population dynamics of the former [10]. In the boreal region, small mammal populations typically show strict cyclicity over long time periods and spanning hundreds of kilometers [10,11,12]. On a smaller spatial scale, the synchrony of cycles has also been documented in western Europe [13] and the boreonemoral region, specifically in the Baltic states [14,15,16]. Based on long-term rodent abundance studies in Fennoscandia [12], substantial evidence for predation being the main reason for small mammal population cyclicity has been gathered [17]. The predation hypothesis suggests that the rodent abundance gradient reflects the relative influence of destabilizing specialists and stabilizing generalists on vole dynamics, modulated by the presence of snow cover [17]. This hypothesis has also been supported by the characteristics of rodent dynamics in central and western Europe [13]. In the late 20th century, rodent cycles showed irregularities and dampening, particularly in the more northern latitudes [18,19,20,21,22]. Due to the large spatial extent, this dampening has been explained by climate forcing—a decrease in delayed density dependence caused by milder winter conditions [23,24,25]. Nonetheless, the generality of this hypothesis has been refuted due to the return of the vole cycle in southern Finland [26].

There is abundant evidence revealing the importance of small mammals, particularly voles, to owls. First, voles form an important part of the owl diet, ranging from more vole-specialized long-eared owl (*Asio otus*, hereafter ASIOTU) [27,28,29,30,31] and Tengmalm’s owl (*Aegolius funereus*, AEGFUN) [32] to more generalist species such as the Eurasian pygmy owl (*Glaucidium passernium*, GLAPAS) [33,34], the Ural owl (*Strix uralensis*, STRURA) [34,35], the tawny owl (*Strix aluco*, STRALU) [28,34,36], and the eagle owl (*Bubo bubo*, BUBBUB) [37]. Second, voles are one of the key elements ensuring higher breeding performance of owls (generally, [6,34]; ASIOTU [29]; AEGFUN [38,39]; GLAPAS [40]; STRURA [41]; STRALU [36]) and survival (ASIOTU [42]; AEGFUN [43]; GLAPAS [44]; STRURA [3,39]; STRALU [36,45]). Finally, vole density affects owl behavior [46,47], migratory decisions [32], and life history via the carry-over effect (events that occur in one season but influence individual success in the following season) [48]. Most of studies on the subject have been conducted in cyclic environments of Fennoscandia, and there have been very few studies conducted in the Baltic states (see [28,36] and references therein). We are not aware of other studies focusing on the boreonemoral region during prolonged periods of depleted small mammal population dynamics.

In this study, we described small mammal population densities in Latvia for the period from 1991 to 2016 and linked them to several population characteristics of six owl species, i.e., ASIOTU, AEGFUN, GLAPAS, STRURA, STRALU, and BUBBUB. First, we described the owl diet and its changes to establish the importance of small mammals and, in particular, voles for different owl species. Then, we divided the owl species based on food niche breadth and the overall proportion of voles in their diet. Next, we compared nationwide owl population trends and their slopes with cyclic vole populations and since dampening. Finally, we described owl breeding performance and related some demographic parameters to the observed population change among owls.

## 2. Materials and Methods

### 2.1. Location and Field Methods

**Figure 1:**
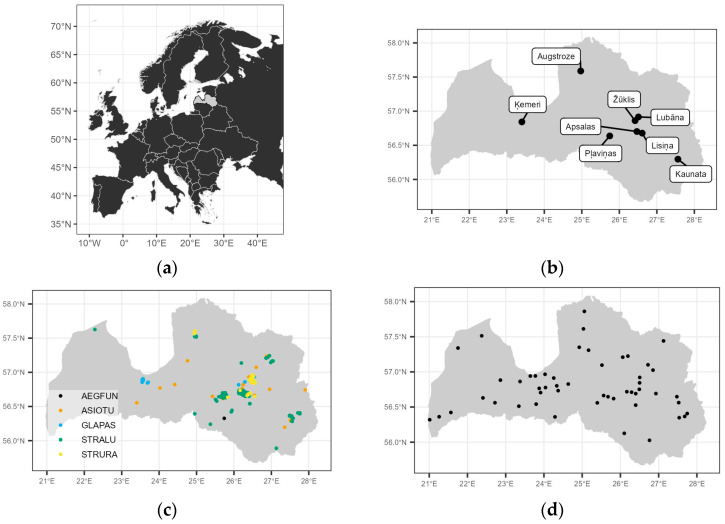
Study site locations: (**a**) Latvia in Europe; (**b**) small mammal monitoring areas; (**c**) owl diet sampling sites; (**d**) owl population change monitoring areas.

This study was conducted in Latvia, northern Europe (Figure 1a). The country is located in the boreonemoral region [49], with a humid continental climate [50]. It lies within the continuous distribution of all six investigated owl species (ASIOTU, AEGFUN, GLAPAS, STRURA, STRALU, and BUBBUB) [51].

#### 2.1.1. Small Mammal Monitoring

Monitoring of the relative abundance of small mammals was conducted with snap traps from 1991 to 2016. It consisted of two schemes: the first with 2 transects per site, with 1 in a forest and 1 in a meadow (1); and the second with 11 transects per site, with 1 in a meadow and 10 in different forest habitats (2).

The first scheme was officially run from 1991 to 2011. A total of 100 snap traps per transect (approx. 5 m between traps) were applied in autumn (August–September) for three to four days [52]. Volunteers partially repeated this monitoring in 2015 and 2016. This scheme was conducted at four sites, but not every site was monitored every year (Table 1).

The second scheme was conducted in autumns (August–September) during the period of 2012–2016 by volunteers. In this scheme, 20–25 snap trap transects (approx. 5 m between traps) were applied in four areas (Table 1), although not all the areas were monitored every year. Forest transects were stratified into 10 categories as follows (minimum rotation ages for dominant tree species in Latvia are provided in Table A1):

- YP—young (clearcuts and stands <7 years old) stands on poor soils;
- YF—young (clearcuts and stands <7 years old) stands on fertile soils;
- MPU—medium-aged (between 8 years and 80% of rotation age) stands on poor soils without drainage;
- MFU—medium-aged (between 8 years and 80% of rotation age) stands on fertile soils without drainage;
- MPD—medium-aged (between 8 years and 80% of rotation age) stands on poor drained soils;
- MFD—medium-aged (between 8 years and 80% of rotation age) stands on fertile drained soils;
- OPU—older (≥80% of rotation age) stands on poor soils without drainage;
- OFU—older (≥80% of rotation age) stands on fertile soils without drainage;
- OPD—older (≥80% of rotation age) stands on poor drained soils;
- OFD—older (≥80% of rotation age) stands on fertile drained soils.

The locations of all the small mammal monitoring sites are shown in Figure 1b.

#### 2.1.2. Owl Diet during the Breeding Season

Owl diet analysis was based on prey remains and pellets found in nests or on the ground near the nest. Only the material from a single breeding occasion was used, based on annual nest-box and cavity inspections (GLAPAS, AEGFUN, STRALU, and STRURA) or based on the assumption that pellets cannot survive for many months in open nests or on the ground in the case of ASIOTU. The material was collected in autumn or winter from nest boxes and cavities and during chick ringing from ASIOTU nests. When collecting material, all the soft contents of nest boxes and cavities were removed. In the case of ASIOTU, all the useable material was collected. The distribution of the owl diet sampling sites in Latvia is shown in Figure 1c.

Analysis of the prey remains and identification of the minimal number of individuals were conducted as described by Vrezec et al. [35]. Insects were assumed to represent 1 g biomass, and amphibians and reptiles were assumed to weigh 16 g based on the average body mass of 100 measured individuals during chick ringing in 2016. In birds, the reference size group (i.e., woodpigeon, song thrush, chaffinch, and chiffchaff) weight from the general literature [53] was utilized. Region-specific weight of mammals from our trapping data or the literature [28,54] was utilized. We assumed young hare *Lepus* sp. to weigh 350 g.

#### 2.1.3. Owl Population Change Monitoring

Monitoring of owl population change was conducted with traditional (territory mapping with playback broadcasting) methods [55,56,57,58,59] in permanent sample areas from 1991 to 2020, as well as with fully standardized point counts (with playback broadcasting) from national Breeding Birds of Prey Monitoring [60] from 2015 to 2022. The spatial distribution of monitoring sites is shown in Figure 1d.

#### 2.1.4. Owl Breeding Performance

We used the number of fledglings per successful nest as a nest-level descriptor of the breeding performance strongly related to food availability. We used four data sources: nest-box inspections (1), information reported by ringers to the Latvian Ringing Centre (2), reports in the Breeding Birds of Prey Monitoring and the previous Monitoring for Owls (3), and citizen scientist reports on nature observation platform dabasdati.lv (4). The first two sources, as well as the third source (partially), covered information from the nests during ringing just before the young fledge. The citizen science and monitoring databases (partially) covered information on the number of young soon after fledging.

Most of the information before 2010 (apart from monitoring data) did not contain exact coordinates to be employed in spatial filtering and removal of duplicate records. Therefore, we combined nest-box inspection, monitoring, and ringing databases based on location attributes (indicated by nest name given by the ringer, which was most often also the person performing monitoring) to remove duplicates. We used citizen science reports only if there was no other information on the species in the particular year in the particular spatial reference. We used the national 1 km projected coordinate grid (epsg: 3059) if coordinates were known or the reported municipality otherwise as a spatial reference.

### 2.2. Data Analysis

We used R software [61] for data analysis, using the ‘tidyverse’ package [62] for data processing and visualizations and ‘sf’ [63] for spatial data. We treated results with *p*-values ≤ 0.05 as statistically significant but also reported insignificant results with full test descriptions.

#### 2.2.1. Small Mammal Monitoring

We standardized the small mammal trapping data to the number of individuals per 100 trap days for further analysis and filtered only for autumn counts due to low representation of spring data. We used a graphical representation of the standardized counts per sampling area and habitat to compare variability between sites and habitats. We used generalized linear mixed-effects modelling (GLMM) to compare the differences in standardized densities between sampling areas, age classes, and soil fertility classes (including meadow habitats as a separate class in the latter two). We created two main effect models per comparison:

- Random intercept per transect and the comparable variable in the fixed part;
- Random intercept per transect and the comparable variable and year as a factor in the fixed part.

We utilized the Poisson family of distribution with a logarithmic link function and selected the best model based on the lowest value of sample-size-corrected Akaike information criterion value (AICc) [64]. We applied marginal means contrasting with Tukey’s *p*-value correction for a post hoc analysis of the comparable variable between groups. For mixed-effects modelling, we implemented R packages ‘lme4′ [65] ‘emmeans’ [66] for contrasting.

We found no differences in either peaks or depressions between the sample areas in graphical analysis or mixed-effects models. Therefore, we used information from all the areas to obtain the countrywide small mammal population change index with TRIM analysis implemented in R package ‘rtrim’ [67]. The baseline model in this tool can generally be written as:

ln(μ_ij_) = α_i_ + β_j_, (1)

where μ_ij_ is an expected count, α_i_ is a site parameter for site i, and β_j_ is a time-point parameter for year j (for full explanation, see [68]). We created a model (model = 3) for the number of individuals pooled across species (small mammals) and separately for voles of genera Microtus and Clethrionomys. We evaluated serial correlation and overdispersion and selected the best model based on the lowest AIC value. We used the multiplicative slope of imputed values reported by TRIM to describe the overall population trend.

We utilized graphical evaluation and Pearson’s correlation analysis to compare the dynamics of both vole groups using yearly indices produced by TRIM analysis. We evaluated the presence of cyclicity with autocorrelation function analysis.

#### 2.2.2. Owl Diet during the Breeding Season

We utilized only samples with at least 5 mammal prey individuals to avoid the influence of some very small samples. We used the small mammal data grouped according to genus as in [69] and other prey groups as described in Section 2.1.2. to calculate Levin’s niche breadth (FNB):

B = 1/∑p_i_^2^, (2)

where p_i_ is the fraction of a given prey in the total consumed biomass [70].

In Supplementary Materials, Table S1, we provide a description of the abundance and weight of the annual diet per owl species. For the description, we used the total number and cumulative biomass of the prey group, their arithmetical mean values, and proportions with Wilson’s 95% confidence intervals.

We utilized linear regression (LM) analysis to evaluate the overall temporal change in food niche breadth. In species with repeated samples from the same nest sites, we also created linear mixed-effects models (LMMs) with nest ID as a random intercept. In the case of singularity in the random part, we employed the result of LM [71,72], as there were too few replicates per nest to contribute to the model fit. Model coefficients in the fixed part did not differ in these models. We fitted simple regression models with niche breadth as a response and year as a regressor variable. Both types of models were parameterized for Gaussian residual distribution with the identity link function.

Then, we evaluated the effect of each previously described small mammal population index on niche breadth with the same LM and LMM approach. We fitted models with the independent variable of the year of breeding, admitting a possibly reduced effect as the small mammal yearly indices represent late summer to early autumn rather than late spring to early summer, when owl breeding occurs.

Additionally, we used the biomass proportion of bank voles and voles of genus Microtus for regression analysis, as well as estimated population indices and bank vole proportion, with the index of Microtus voles. In this set of analyses, we applied GLMMs with a random intercept for nest ID and generalized linear models (GLMs) if the identifier did not contribute to model performance due to the number of replicates being too low [71,72]. We used the binomial family of distribution with logistic link function to compare the proportion of prey in the diet with its annual population index value in nature the same year.

Due to the small number of samples, we only described the AEGFUN diet without further analysis.

#### 2.2.3. Owl Population Change Monitoring

Owl population monitoring started in 1990, but not all species had reliable information since the beginning of the period. Due to its preference for large forest massifs, STRURA was covered only since 1993. Due to a lack of knowledge on the monitoring of GLAPAS, its population change can be analyzed only since 2004. Due to the low population size, we did not have reliable data on the population change of BUBBUB.

We combined the data from both owl monitoring schemes if sites had all the planned census activities—for sample areas, sufficient coverage marked by an observer and for point count sites, four standardized visits to each point every year. To analyze population change, we drew on TRIM as described in Section 2.2.1. We exploited only the sites with information from at least two years and comparable effort according to the prerequisites [73].

We further calculated yearly indices and overall population change, as described by multiplicative slope with standard error [68,74], covering the whole available data period for the species. Then, we calculated two different slopes with relatively cyclic small mammal populations and since cycles had vanished. We used 2004 as the threshold for this division because it is:

- The approximate time since when the small mammal populations did not recover to previous peaks;
- The approximate midpoint of small mammal monitoring;
- The approximate midpoint of STRURA monitoring;
- The beginning of GLAPAS monitoring.

To calculate “before” and “after” trends, we selected the necessary parts of yearly indices and conducted linear regression on ln-transformed indices. We defined index 1991–2004 as “before” and index 2004–2016 as “after”. To obtain significance tests, we transformed the time to start with year 1 in each group to use in regression with interaction between time and period. We defined “before” as a reference level.

#### 2.2.4. Owl Breeding Performance

We calculated the annual mean values of spatially cleaned results because in most cases, we were not able to match different breeding performance reports to an exact breeding location (nest or territory). To provide more generalizable information, we calculated bootstrapped 95% confidence intervals from 1000 bootstrap resamples and implemented them in visualization. We used the annual mean values to establish a temporal trend of overall change in breeding performance. We employed slightly different approaches for further processing because the amount of information varied among owl species.

For the two species (STRALU and STRURA) with most data available on a nearly annual basis, we compared the temporal trends before and after the dampening of small mammal cycles in 2004. We used Gaussian linear regression with the annual mean number of fledglings as a response and compared the trends between the periods as in population change analysis.

We used all the years available to correlate the annual mean breeding performance of STRALU and STRURA with small mammal population indices in the year of breeding and one year before to evaluate a possible carry-over effect. In the case of ASIOTU and BUBBUB, we used a reduced timeframe (from 2002 and 2001, respectively) to avoid possible artefacts due to irregular sampling and a small sample size. We used Spearman’s rank correlation analysis due to some outliers and a slightly curved scatter plot.

We harnessed the R package ‘Hmisc’ [75] for bootstrapping and base R for correlation and regression analysis.

## 3. Results

### 3.1. Small Mammal Monitoring

**Figure 2:**
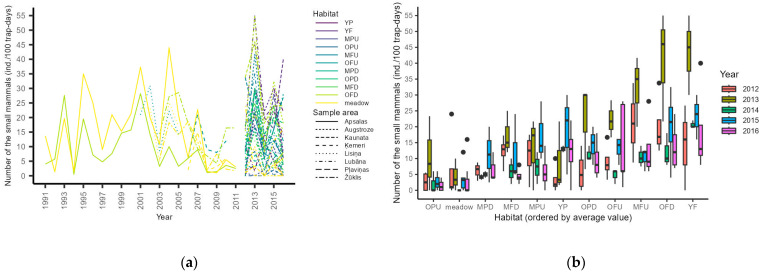
Observed small mammal (pooled across species) population densities per 100 trap days: (**a**) variation over time (1991–2016) in different sample areas and habitats; (**b**) differences in the observed number of small mammals (per 100 trap days) between habitats over time (2012–2016).

The number of small mammals per 100 trap days over time in different sample areas and habitats is illustrated in Figure 2a. As the figure depicts, the peaks and depressions matched well between areas over time, with only a slight stochasticity between habitats within the same areas. This was confirmed by GLMM analysis, showing no significant differences in marginal mean ratios of the number of small mammals per 100 trap days among sample areas when accounting for an individual transect in a particular year (Table A2). There were observable differences in the relative abundances of small mammals between habitats (Figure 2b). GLMM analysis revealed that meadow habitats had significantly lower abundances than any forest age group but with no differences between age groups (Table A3). Comparison of fertility groups revealed that meadows had significantly lower abundance and that forests on fertile soils had significantly higher abundance after accounting for multiple comparisons (Table A4). In every comparison, GLMM including the hierarchical random intercept of transect in year and only the variable of interest in the fixed part was the best-performing model (with the lowest AICc values).

**Figure 3:**
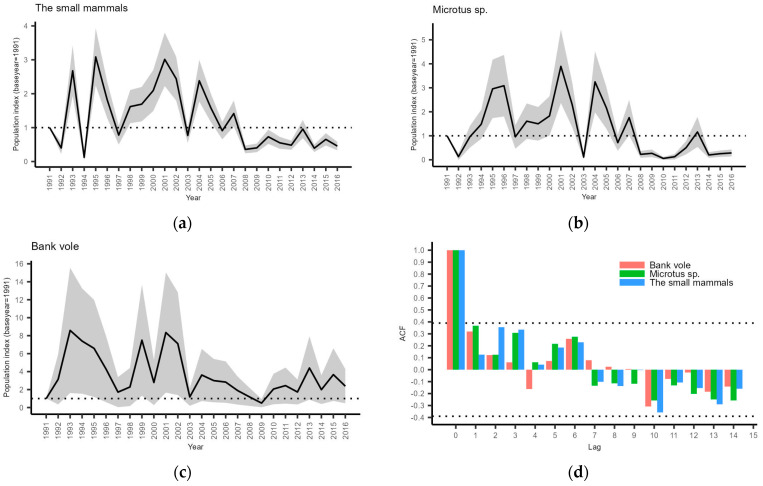
Population indices with standard errors: (**a**) pooled small mammals; (**b**) voles of genus Microtus; (**c**) bank voles; and (**d**) autocorrelation function analysis of the yearly small mammal indices; dotted lines indicate the threshold of significance.

We pooled all the results to conduct a population change analysis because there were no important differences between the areas. All three models suggest statistically significantly declining populations (total number of small mammals: S = 0.9671 ± 0.0083, *p* = 0.0007; Microtus voles: S = 0.9306 ± 0.0167, *p* = 0.0005; bank voles (*Clethrionomys glareolus*): S = 0.9706 ± 0.0128, *p* = 0.0325). Yearly abundance indices are shown in Figure 3a–c.

Some cyclicity in populations was apparent in the late 20th century and early 21st century, but it lost amplitude later (Figure 3a–c). Since 2008, the total number of small mammals (Figure 3a), as well as Microtus voles (Figure 3b), has remained relatively stable and at low density level. The same happened with bank voles (Figure 3c) earlier—around 2003. Graphical evaluation indicated a certain degree of matching between vole population indices in depression years, e.g., 1997, 2003, and, to some extent, 2014, with overall moderate (r = 0.5604) and statistically significant (*t* (24) = 3.3146, *p* = 0.0029) correlation. Autocorrelation function analysis showed no temporal cyclicity in any species group (Figure 3d).

### 3.2. Owl Breeding Season Diet

**Figure 4:**
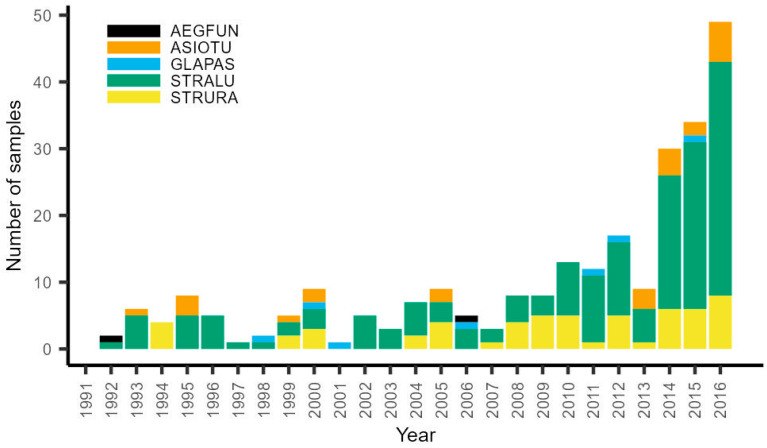
Number of owl diet samples (with at least five mammal individuals) over time.

In total, 164 STRALU samples from 86 different locations covering 23 years, 56 STRURA samples from 38 different locations covering 15 years, 24 ASIOTU samples from 21 different locations covering 9 years, 7 GLAPAS samples from 7 different locations covering 7 years, and 2 AEGFUN samples from 2 different locations covering 2 years (Figure 4, Table S1) were analyzed. The description of annual food composition per species is presented in Supplementary Materials, Table S1.

**Figure 5:**
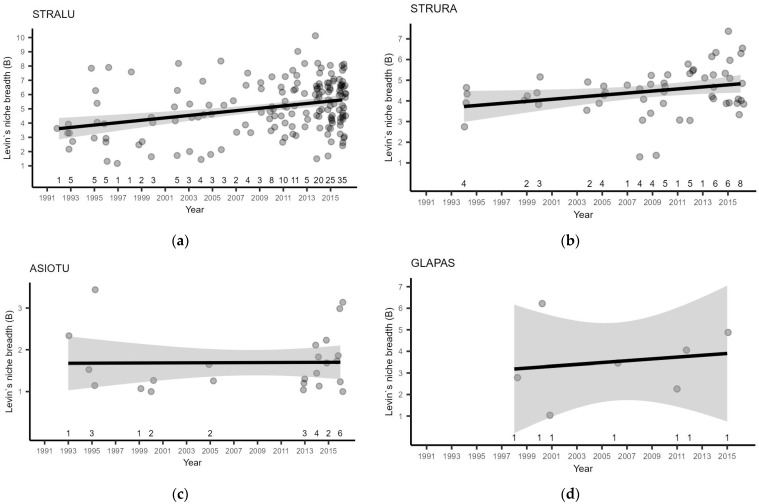
Temporal change in Levin’s food niche breadth in four owl species: (**a**) STRALU; (**b**) STRURA; (**c**) ASIOTU; (**d**) GLAPAS. Grey points represent individual prey samples. The black line is the linear regression trend, and the grey ribbon is the 95% confidence interval. Numbers above the X-axis represent the number of samples. The Y-axis range differs depending on the facet.

The overall average food niche breadth (FNB) of STRALU was 5.125 (95% bootstrapped confidence interval (bCI), 4.867–5.423). FNB increased significantly (β = 0.0840 ± 0.0198; *t* (129.2401) = 4.249; *p* < 0.0001) from 1992 to 2016. There were notable differences between samples in any given year (Figure 5a), and inclusion of nest ID as a random effect provided some help in dispersion taming (LMM: R^2^_conditional_ = 0.115, R^2^_marginal_ = 0.101, ICC = 0.015), indicating some degree of territory-specific variability. Although the explained variances were low, we found a statistically significant negative effect of each of the small mammal population indices on FNB (Table 2). The proportion of voles (both groups) in owl prey correlated positively with the vole abundance indices, but STRALU showed preference for Microtus voles, as their abundance index had a significant negative correlation with the bank vole proportion in prey (Table 3). On average, Microtus voles accounted for 15.55% of biomass, whereas bank voles and voles in total accounted for 5.51% and 31.65% of biomass, respectively.

STRURA also showed large variability in the diet, as overall FNB was 4.485 (95% bCI, 4.201–4.758). We found a significant (β = 0.0499 ± 0.0227; *t* (54) = 2.194; *p* = 0.0325) increase in FNB from 1994 to 2016 (Figure 5). The overall variability was lower than in STRALU, but no nest-specific intercepts were found to improve the model, and LM could explain only about 6.5% of the variance (R^2^_adj._ = 0.06486). We found no correlation between FNB and small mammal population indices (Table 2). The species showed a strong preference for Microtus voles, the proportion of which in prey correlated positively with its abundance index, while higher abundance in nature led to a lower proportion of bank voles in prey (Table 3). We suggest preference as a reason for the negative correlation of the bank vole abundance index with its proportion in prey because both vole abundance indices were correlated (Table 3). On average, Microtus voles accounted for 15.10% of biomass, whereas bank voles and voles in total accounted for 6.07% and 31.78% of biomass, respectively.

The overall FNB of GLAPAS was 3.526 (95% bCI 2.355–4.756), showing a temporal increase (Figure 5). However, this increase was not found to be statistically significant (LM: β = 0.0420 ± 0.1136, *t* (5) = 0.369, *p* = 0.727; R^2^_adj._ = −0.1681), probably due to high intersample variability and small overall sample size. We found no correlation between FNB and small mammal population indices (Table 2). Results of prey proportion and relative abundance in nature were similar to those for STRURA, but the preference for Microtus voles was greater, serving as better explanator of the bank vole proportion in prey (Table 3). On average, Microtus voles accounted for 10.03% of biomass, whereas bank voles and voles in total accounted for 11.31% and 29.46% of biomass, respectively.

ASIOTU’s FNB was only 1.629 (95% bCI, 1.429–2.002), and LM did not suggest any temporal change (β = 0.0010 ± 0.0176, *t* (22) = 0.059, *p* = 0.953; R^2^_adj._ = −0.0453). We found no correlation between FNB and small mammal population indices (Table 2). The species showed a strong preference for Microtus voles over bank voles, and Microtus vole abundance in nature significantly correlated with its proportion in prey (Table 3). On average, Microtus voles accounted for 71.18%, whereas bank voles and voles in total accounted for 4.64% and 87.13% of biomass, respectively.

The two analyzed samples of AEGFUN had FNBs of 1.588 and 4.318, respectively (Table S1). On average, Microtus voles accounted for 62.89% of biomass, whereas bank voles and voles in total accounted for 13.81% and 76.69% of biomass, respectively.

### 3.3. Owl Population Change

**Figure 6:**
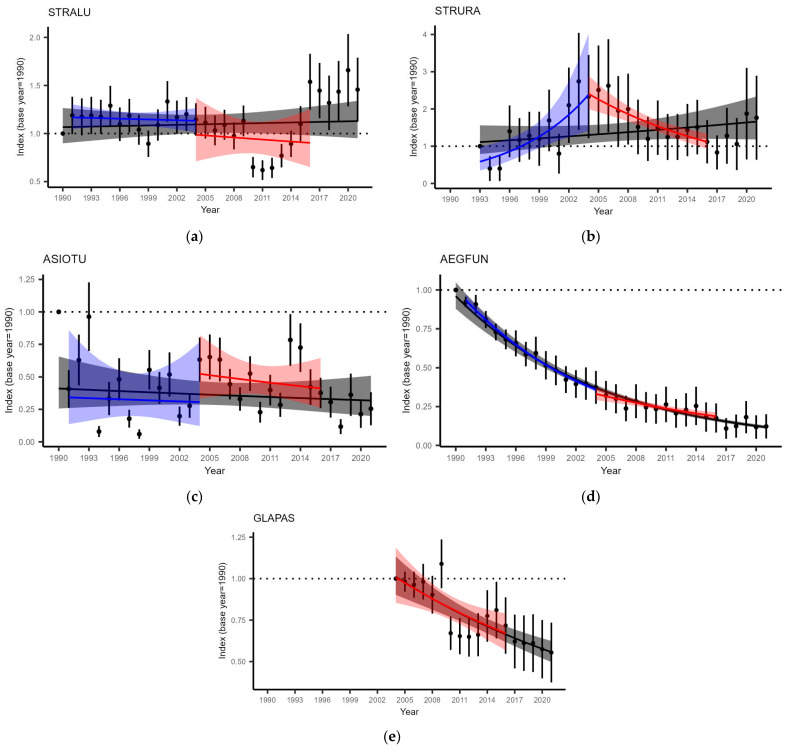
Population changes in owl species: (**a**) STRALU; (**b**) STRURA; (**c**) ASIOTU; (**d**) AEGFUN; and (**e**) GLAPAS. Black dots with error bars are yearly indices with standard errors, and trend lines and ribbons with different colors represent different population trends: black—overall trend, blue—1991–2004, and red—2004–2016. The *Y*-axis range differs depending on the facet.

Our results differed between species when comparing owl population changes before and after small mammal cycle depletion (Figure 6, Table 4).

STRALU, with an overall (1990–2021) stable population (S = 1.002 ± 0.005), exhibited no significant difference in population trends before and after depletion (Table 4). Although the population experienced a considerable depression during the period of 2010–2012, it has since recovered (Figure 6a).

We obtained similar results for ASIOTU, with an overall (1990–2021) stable population (S = 0.992 ± 0.010) and no significant difference between the periods (Table 4). However, a visual extension of the trend since mammal population depletion suggested a decline that might be obscured by fluctuating population (Figure 6c).

The results of STRURA were different; although overall (1993–2021), the population was classified as stable (S = 1.014 ± 0.012), there was a significant difference in trends (Table 4). This species had a strongly increasing population before 2004 and a steep decline since small mammal depletion (Figure 6b).

Population change information for GLAPAS was available only since the depletion, and its overall (2004–2021) negative population trend (S = 0.965 ± 0.017) was similar to that observed in 2004–2016, reflecting a significant decline (Figure 6e, Table 4).

The results for AEGFUN are interesting, as the overall (1990–2021) population had a steep decline (S = 0.934 ± 0.020) that fit with estimated yearly indices (Figure 6d). Nevertheless, the difference between slopes “before” and “after” depletion was significant (Table 4) and suggests a steeper decline during the pronounced small mammal dynamics than since the depletion of cycles. However, visually extending the trajectory of “after” revealed a pattern to that in the “before” period; thus, the difference could be an artefact due to an increased influence of some years.

### 3.4. Owl Breeding Performance

**Figure 7:**
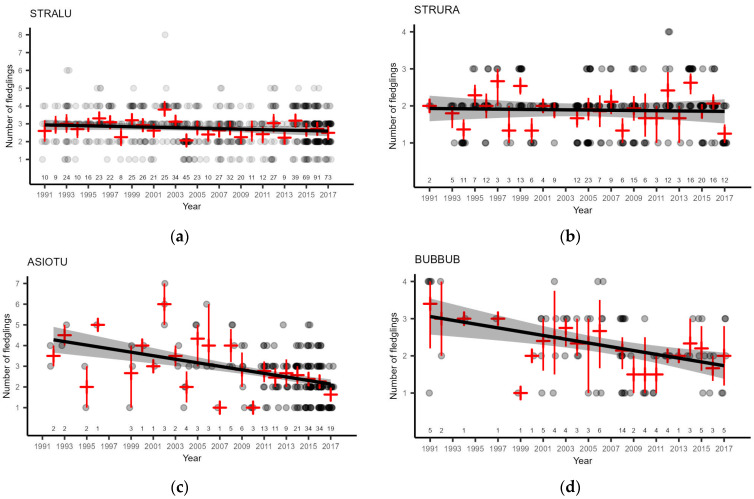
Annual number of fledglings of four owl species: (**a**) STRALU; (**b**) STRURA; (**c**) ASIOTU; (**d**) BUBBUB. Grey points represent individual nest performance. Red crosses are bootstrapped 95% confidence intervals around the annual mean value. The black line is the linear regression through mean values, and the grey ribbon is the 95% confidence interval. Numbers above the X-axis represent the annual number of samples. The *Y*-axis range differs depending on the facet.

On average, STRALU had 2.32 (95% bCI 2.23–2.40; *n* = 934) fledglings per successful nest. Despite the appearance of reduced breeding performance since 2004 (Figure 7a), these differences were not statistically significant (Table 5). The overall trend of breeding performance was insignificant (β: −0.0041 ± 0.0088, *t* (26) = −0.467, *p* = 0.645; R^2^_adj._ = −0.0298; F (1;26) = 0.2179, *p* = 0.6445). We did not find a correlation with the small mammal abundance indices in the year of breeding or the previous autumn (Table 6).

On average, STRURA had 1.69 (95% bCI 1.58–1.80; *n* = 280) fledglings per successful nest. Despite the appearance of some differences in trends of breeding performance before and after small mammal cycle dampening (Figure 7b), they were not statistically significant (Table 5). The overall trend of breeding performance was insignificant (β: −0.0014 ± 0.0122, *t* (24) = 0.112, *p* = 0.912; R^2^_adj._ = −0.0411; F (1;26) = 0.0125, *p* = 0.912). We did not find correlations with the small mammal abundance indices in the year of breeding or the previous autumn (Table 6).

The average number of ASIOTU fledglings was 2.54 (95% bCI 2.38–2.72; *n* = 189) per successful nest. However, this population parameter declined over time (Figure 7c) by approximately one fledgling in 16 years (β: −0.0627 ± 0.0297, *t* (22) = −2.109, *p* = 0.0466; R^2^_adj._ = 0.1303; F (1;22) = 4.446, *p* = 0.0466). We did not find a correlation with the small mammal abundance in the year of breeding or the previous autumn (Table 6).

The average number of BUBBUB fledglings was 2.26 (95% bCI 2.04–2.46; *n* = 81) per successful nest. This population parameter declined over time (Figure 7c) by approximately one fledgling in 22 years (β: −0.0450 ± 0.0137, *t* (20) = −3.293, *p* = 0.0036; R^2^_adj._ = 0.3191; F (1;20) = 10.840, *p* = 0.00036). We found positive correlations with the abundance index of pooled small mammals in the breeding season and the previous autumn, with a stronger effect of the latter (Table 6). The effect of the Microtus vole abundance index in the previous autumn was also statistically significant and positive (Table 6).

We had too few reliable observations of GLAPAS and AEGFUN breeding performance for analysis; therefore, we provide only a description of the average values: r = 2.75 (95% bCI 2.00–3.50; *n* = 8) and 1.75 (95% bCI 0.50–2.75; *n* = 4), respectively.

## 4. Discussion

### 4.1. Small Mammal Monitoring

Overall, the small mammal densities and trapping indices in our study (Figure 2) were similar to findings in the neighboring countries of Estonia [16,76] and Lithuania [77,78,79]. We found that meadow habitats had lower abundance of small mammals than forests (Table A3, Table A4). However, due to large within-class variation, no clear differences between age groups were found (Table A3). In Estonia [16] and Lithuania [78], increasing small mammal abundance has been recorded with increasing forest age in early meadow–forest succession. Additionally, studies from Finland [80] and Norway [81] reported that mature forests had the highest abundance of voles.

However, a study conducted in northern Sweden suggested that young stands have higher small mammal diversity and abundance if a large amount of felling remains is left [82]. Many authors have found that high vegetation complexity, habitat structural diversity, and abundance of coarse woody debris are important factors that can help to ensure high diversity and abundance of small mammal species in young stands and unmanaged habitats under natural succession [79,81,82,83,84,85].

The negative effects of intensive forestry have been found to be important at the landscape scale [83,84,85,86]. However, in a mosaic landscape, ecotones (with at least 100m buffer zone of habitat edges) have been found to contain the highest small mammal density and diversity [77,81,84].

Although the insufficient number of transects and occurrences in our study did not allow for statistical testing of forestry impacts, we found some declines in the small mammal numbers linked to forestry activities but unrelated to changes in other transects (Figure 2). Most of the small mammal monitoring transects were in intensively managed forests. However, two of the longer-term areas were in protected areas (Apasalas and Žūklis) and also showed dampening of the cycles, suggesting larger than local (or management) effects on the dampening of the cycles.

One of the most robust explanations of cyclicity was provided by Hanksi et al. [17] with further extensions for different systems (see [87] for overview). One of those extensions, modelling multispecies rodent assemblages, revealed transient dynamics that alternated between long time periods with cyclic and non-cyclic fluctuations [19]. These fluctuations were expected to cover relatively small spatial scales, yet the phenomenon of dampened cycles was more recently found to occur Europe-wide [25], suggesting broader environmental drivers, for example, climate change [22,23]. However, in some parts of Europe, the period of dampened vole population cycles has been shorter than in others, refuting the generality of the climate forcing hypothesis [26]. Our results also showed clearly dampened vole cycles in Latvia (Figure 3a–c). The fact that such a dampening of cycles has not been reported in neighboring counties [14,15,16] suggests some smaller-scale processes, such as those described by predator–prey models. Although Hanski et al.’s models were created for the Fennoscandian environment, their generality has also been shown in central and western Europe [13]. According to these models and previous studies (see [87] for an overview), generalist predators tend to stabilize rodent dynamics, and nomadic avian predators have a similar effect on rodents, although they also increase the regional synchrony, whereas specialist predators have been thought to maintain the fairly regular rodent cycles [19].

### 4.2. Numerical Response of Owls

We found a statistically significant relationship between the proportion of voles (Microtus and bank voles) in owl diet and their relative abundance indices in nature (Table 3). This means that although we drew on mammal abundance information from autumn, it was still able to represent their abundance in owl prey. It is known that small mammal densities increase during summer [52] and that spring counts represent winter survival and reproduction [88], but the relative value of the current year (spring or autumn) still represents part of the cycle in cyclic environments [41]. We found preference for Microtus voles in every analyzed owl species in terms of the proportion in owl diet; these voles also accounted for a higher biomass proportion than bank voles (Table 3). Generally, a higher proportion of Microtus voles than bank voles in owl diet can be related to different breeding biology of voles and dispersal between vole species groups and predator-escaping behavior (see [19] for an overview).

#### 4.2.1. Long-Eared Owl

We found ASIOTU to have the narrowest FNB among the investigated species. The calculated values were slightly lower than in Lithuania [28], possibly due to the pooling of the results to genus level. This species is known to be a small-mammal specialist in Europe [27] with a high proportion of Microtus voles in their diet [28,89,90,91,92,93]. ASIOTU has been found to show strong functional responses of diet, breeding success, and dispersal to vole abundance [29,30,31,42]. It has even been suggested that species can adapt migratory behavior and breeding region selection during migration to account for vole abundance [94]. Moreover, this species may even exhibit repeated breeding attempts if the vole abundance is high [95,96].

Although the average breeding performance in Latvia was similar to the 2.94 ± 0.42 (μ ± SD; *n* = 1339) recorded in Finland [97] and 2.39 (*n* = 72) in the United Kingdom [95], we observed a significant decline in the number of fledglings per successful nest, i.e., more than one chick in three generations (5.7 years; [98]). The steepest decline occurred in the last two generations and matched the time of dampened populations of small mammals (Figure 7c). The declining breeding performance did not have an impact on the population change (2004–2016), but extension of the trend (2004–2021) showed a significant decline (β: −0.0530 ± 0.0191, *t* (16) = −2.772, *p* = 0.0136). We suspect that for a longer period of time, the ASIOTU population was supported by immigration of migrating individuals hatched elsewhere [29,90,99,100] and that a later decline implies a delayed response of returning individuals of Latvian origin.

Habitat composition and prey abundance have been found to be the most important factors shaping local ASIOTU populations [90,101,102,103] because the species shows no strong territorial defence and hunting grounds may largely overlap between neighboring pairs [104,105]. We found no correlation between the breeding performance and prey abundance indices in the year of breeding or the previous year (Table 6), likely due to selection of breeding territories with sufficient abundance of prey. This is supported by the knowledge of species benefitting from relatively small landscape elements, for example, flower strips [103].

#### 4.2.2. Tengmalm’s Owl

Although we had only two samples of AEGFUN diet, its FNB suggested a high specialization, which was supported by a high proportion of voles in the diet. The observed proportions in Europe have shown a high importance of voles (overview in [32]), averaging 54.89% according to previous studies. This species has a strong functional response to vole abundance influencing habitat selection via hunting behavior [47,106,107,108], the timing of breeding and breeding performance [6,39], and survival [39,43]. Even with a certain degree of carry-over effect, the species has shown strong adaptability to fluctuating food conditions in terms of breeding performance [38].

The average long-term number of fledglings in Finland is 4.04 ± 0.62 (μ ± SD; *n* = 13,817) [38] and around 2 fledglings per successful nest in poor vole years [32,109]. The scarcely available data on the breeding performance in Latvia suggests that it is similar to that in vole depression years in Finland.

We found a steep decline of AEGFUN population throughout the studied period (Figure 6d), but it was slower with depleted population dynamics of small mammals (Table 4). We expected this to be an artefact of some better seasons or immigration from Fennoscandia and Russia [32] rather than an actual difference; therefore, we extended the period of analysis in the “after” group. Our results (β: −0.0618 ± 0.0074, *t* (16) = −8.302, *p* = <0.0001) showed a decline since 2004 closely matching the overall population decline and the slope of the period with pronounced population dynamics of small mammals. Some researchers have hypothesized potentially negative effects of increasing STRURA population on the AEGFUN population [32]. However, we did not find any AEGFUN as a prey of STRURA, although superpredation is known [34] and both species coexisted in the same study areas (authors’ personal observations). Furthermore, in central Europe, breeding in the proximity of STRURA has been found to protect AEGFUN against STRALU [110,111,112].

Population declines have also been reported in Finland, Sweden, and Estonia [113], suggesting larger-scale factors affecting the population. This species is a mature spruce and mixed forest specialist [47,101,108,114,115,116,117,118,119]. These are habitats with some of the highest densities of small mammals [79,80,81,82,83,84,85]. We consider the loss of species-specific habitats to be the most important factor in population decline, amplified by dampened dynamics of small mammal populations in Latvia. The forestry intensity, as measured by tree cover loss, is increasing in Latvia and, in particular, in priority sites for species conservation [120].

#### 4.2.3. Eurasian Pygmy Owl

We found average level of specialization of GLAPAS and the preference for Microtus voles was strongest among the analyzed owl species (Table 3), although with a low proportion of voles in the diet. The vole proportion was similar to the breeding season diet in Finland [34] and in central Europe [121]. Masoero et al. found a strong numeric and functional response of GLAPAS to vole abundance in winter [33], suggesting not only age- and gender-specific preference for voles but also stronger migratory behavior during low-vole-density years in boreal Finland. During the years of higher vole population densities, breeding density and performance of GLAPAS also increase [6,122]. The dependency on voles has been found to be stronger in boreal than boreonemoral regions, with breeding both in low and peak vole years in the latter [40]. In the boreonemoral zone, the onset of breeding was later with no correlation with breeding performance, and the clutches were slightly smaller than in the boreal zone [40].

For the few documented records of successful breeding in Latvia, the values were markedly lower than 5.85 ± 0.55 (μ ± SD; *n* = 13,817) in Finland [97] and boreal Norway (6.9 ± 1.1) and somewhat lower than in boreonemoral Norway in vole crash years (3.7 ± 2.8) [40]. The difference relative to boreonemoral Norway indicates a possible cumulative effect of longer-term dampened population cycles of small mammals, which is supported by a declining GLAPAS population. The population in Estonia and Lithuania is increasing [113], but it is declining in Latvia (Figure 6e and Table 4) and in Finland [97,113]. It has to be noted that only Finland and Latvia were able to provide analytical assessment of the population (type: interval) in the last report on the Article 12 of the Birds Directive [113]; therefore, it cannot be ruled out, that the increase in the other Baltic states is more based on increased survey efforts and knowledge than a genuine change. Although irruptions linked with low rodent availability occur from time to time [123], it is unlikely that Finland and Latvia are source populations for neighboring countries with declining populations themselves, despite the increased distribution of the species [51]. This is supported by similar patterns of yearly indices in Latvia (Figure 6e) and Finland [97] but with a steeper decline in Latvia.

GLAPAS is known to be a structurally rich, mature spruce and mixed forest specialist species during the breeding season [122,124,125,126,127,128,129,130,131], and clearcuts and logging have been shown to affect habitat suitability [131], as well as population size [132]. Structurally rich mature forests are habitats with some of the highest densities of small mammals [79,80,81,82,83,84,85,133]. Latvia and Finland are the countries in Europe with the highest forestry activity, even in protected areas [134]. We suspect the loss of species-specific habitats to be the most important factor in the population decline, amplified by the dampened dynamics of small mammal populations in Latvia. The forestry intensity, as measured by tree cover loss, is increasing in Latvia and, particularly in priority sites for species conservation [120].

#### 4.2.4. Ural Owl

One of the highest and temporarily increasing FNB values was found in STRURA. We found a low proportion of voles in the diet of this species. This proportion, when compared by count, was lower than in Finland [34,35,135,136], similar to that in Belarus [137,138,139], and higher than that in Slovenia [35]. When comparing the food niche as a whole, STRURA diet in Latvia was found to be similar to that in Finland during the low vole phase [35]. Although this species is known to be a generalist predator, a strong functional response to vole abundance has been proven in Fennoscandia, ranging from the timing of breeding and breeding performance [3,6,39,41,140] to winter survival [3,6,39,41,45] and even demonstrating a carry-over effect from the previous year [48] and a change in behavior [46,141,142].

Not only the food niche but also the breeding performance of STRURA in Latvia was similar to that in Finland in bad vole years. In Latvia, we observed, on average, 1.69 (95% bCI, 1.58–1.80; *n* = 280) fledglings per successful nest and no temporal trend. The corresponding overall value in Finland (1986–2016) is 2.59 (±0.43 SD, *n* = 18,901; [98]) and between 1.3 and 2 [3,41] in bad vole years, roughly matching our results. Given the strong numerical response to voles, we expected a declining trend in breeding performance, but we did not find it. We consider this an example of strong parental investment [142,143,144] as reflected by adjustments in hunting activity and possibly habitat selection [101], demonstrating the high plasticity of the species [35]. As Figure 2 shows, even with dampened small mammal cycles, there are habitats with high prey abundance, allowing prey to meet the demands of the young. The size of nest boxes in Latvia is similar to that in Finland [35] and cannot be suspected as a reason for lower breeding performance.

Increasing STRURA populations and expanding range, even increasing the niche of utilized habitats, was observed in many parts of Europe during the first decade of the 21st century [145,146,147,148]. This overlaps with the increase in Latvia and breeding occurrences in a mosaic landscape [101]. Given the extent of population increase, some unknown large-scale factors are most likely the explanation. Nevertheless, in Latvia, the period of steep decline in the species population overlapped with the dampening of the small mammal population dynamics. We consider the relative abundance of small mammals to be an important collider for overall habitat change, as species ecological niche analysis in Latvia suggests strong dependency on large forest massifs with dominance of mature forests and only some openings [102]. These are habitats found to be important for the same species elsewhere [146,149,150]. Although the range is still expanding in Latvia [151], the overall population size is declining [113]. The forestry intensity, as measured by tree cover loss, is increasing in Latvia, in particular in priority sites for species conservation [120]. We consider this as an argument for the conservation of mature forests important for the species and holding higher densities of its main prey, i.e., small mammals [81].

#### 4.2.5. Tawny Owl

The highest FNB value was found in STRALU with a relatively low proportion of voles in the diet. The average FNB value was slightly lower than in Lithuania [28]. We observed a temporal increase in FNB, which was similar to the observation in Lithuania, with a declining proportion of Microtus voles [36]. This species is known to be a generalist [152]. Its food composition can considerably vary between breeding regions within the same year and between years in the same breeding territory [28,36,152,153,154,155,156,157,158,159,160]. Nevertheless, in the cyclic environment of Fennoscandia, a strong numerical response to vole abundance has been reported, including the timing of breeding [6,160], breeding performance [3,5,6,39,160,161], and winter survival [3,39].

Both the population change and breeding performance of STRALU were stable and showed no differences relative to pronounced and dampened vole cycles. Breeding performance was lower than 3.26 ± 0.41 (μ ± SD; *n* = 9668) in Finland, where the population was also stable [97], as well as in Lithuania, where an increasing trend of breeding performance (2002–2014) was observed and co-occurred with a decline in the number of breeding pairs [36]. We consider the relatively low breeding performance in Latvia to be related to the high population density (estimated to be around 16,604 in Latvia and below 4000 in Lithuania) [113]. The observed depression of the STRALU population from 2010 to 2012 in Latvia partially matched with Lithuania [36]. This was likely the consequence of two consecutive snow-rich winters, with multiple freeze—thaw events forming ice sheets in snow cover—factors reducing species survival [45,162,163]. This event did not affect breeding performance, and the population recovered quickly.

We think that the quick population recovery and overall stable breeding performance, even with increasing FNB values, was possibly due to the breeding habitat availability. Although this species is a well known generalist breeding from cities to large forest massifs in more southern latitudes [34], in the boreonemoral region, STRALU has been found to prefer forest edges over the interior [101,164]. With increasing forestry and forest fragmentation, more suitable landscapes for this species are created [101], probably overwhelming the negative effects of depleted small mammal populations.

#### 4.2.6. Eagle Owl

The largest European owl species, BUBBUB, is known to be a generalist predator, with the proportion of rodents in its diet ranging from 0 to 97.7%, with average of 49.7% among 182 studies (overview in [37]). We do not have reliable information on the diet of BUBBUB in Latvia, but during the ringing of the young, many bird feathers are found, as most of the known breeding sites are in close proximity to waterfowl lakes and landfills [101]. This species is resident with no known seasonal migrations in Europe [37,165], and breeding dispersal occurs mostly due to the loss of a mate [37]. We speculate that the BUBBUB population in Latvia depends highly on voles, at least in winter, when bodies of open water are typically frozen and most waterfowl and gulls in Latvia have moved to wintering areas [151]. This is supported by a study conducted in Finland evaluating the robustness of the alternative prey hypothesis for BUBBUB [166]. A correlation was found between vole abundance in nature and their proportion in the diet, and the proportion of alternative prey was found to be nearly independent of its abundance in the field [166]. Several other studies highlight the high proportion of voles in the BUBBUB diet [167,168,169].

The overall average number of fledglings per successful nest in Latvia was similar to that in Europe—around two (overview in [37]); however, we observed a declining trend, with a loss of more than one fledgling in two generations (generation length is 12.1 years [170]). We found that breeding performance was correlated with the abundance index of small mammals in field, and the correlation with the value from the previous autumn was stronger (Table 6). Other studies found that BUBBUB pairs with a diet based on high-value foods (rabbits and rodents) have comparatively larger broods and breed earlier [171,172], and higher reproductive productivity was associated with a higher proportion of the main prey (rats and rabbits in Spain) in the diet [173]. Our data do not lend themselves to examining such a relationship between breeding performance and diet. Nevertheless, we assume that the negative effect of a reduced abundance of small mammals highlights a carry-over effect, influencing adult fitness in spring and thereby reducing the breeding performance. This phenomenon is well known in STRURA [41,48,143,174,175] and has proven to be of increasing importance with the size of an owl species in Finland [6]. Ecological niche analysis in Latvia also suggests the importance of habitats with higher vole abundance [81] for BUBBUB [101].

Although we had annual data on a limited number of nests, they formed an important part of the whole population estimated at around 24 breeding territories, indicating a declining national population trend [113]. We consider our findings of a possible carry-over effect to be important in species conservation and to be linked to population decline via reduced breeding performance, as well as reduced winter survival, as both should be related via fitness, although this relationship needs to be studied more directly. Nevertheless, we consider the conservation of habitats important for breeding and winter feeding, together with nest site protection from ground predators, which is necessary to reduce the effects of dampened population dynamics of small mammals.

## 5. Conclusions

1. Small mammal relative abundance indices have shown depleted population cycles since approx. 2004. This has impacted the breeding performance, food niche breadth, and population trends of owl species to various degrees depending on the particular species;
2. The number of ASIOTU fledglings has declined since the depletion of small mammal populations. The population size of the species declined later and was significant for the period from 2004 to 2021. ASIOTU is the most specialized of the analyzed owl species in terms of the proportion of voles in the diet;
3. The breeding performance of the three forest specialist species AEGFUN, GLAPAS, and STRURA in Latvia was similar to vole depression years in the boreal and boreonemoral regions;
4. Populations of GLAPAS and AEGFUN declined in Latvia and showed no difference compared to periods with pronounced or depleted population dynamics of small mammals. In contrast, the population of STRURA has shown a significant decline since rodent depression. We suggest the depletion of the small mammal population dynamics to be an important negative contributing factor to more important effects of forestry, although the impact of forestry needs to be investigated further;
5. Neither the breeding performance nor population size of STRALU changed between the compared periods with pronounced and depleted population dynamics of small mammals. This suggests a strong plasticity of the species, as food niche breadth was temporarily increased;
6. We found evidence that suggests the dependency of BUBBUB on voles via a carry-over effect. The breeding performance of BUBBUB was significantly correlated with the abundance indices of small mammals in nature in the previous autumn.

## Figures and Tables

**Table 1:** Descriptions of small mammal monitoring efforts.

Sample Area	Period	Description
Apsalas	1991–2011; 2015–2016	2 habitats: meadow and forest (OFD); 100 traps per transect
Lisiņa	2001–2005	2 habitats: meadow and forest (OFU); 100 traps per transect
Žūklis	2003–2011; 2015–2016	2 habitats: meadow and forest (OFD); 100 traps per transect
Ķemeri	2006–2010; 2015–2016	2 habitats: meadow and forest (OFU); 100 traps per transect
Kaunata	2012–2016	11 habitats: 1 meadow and 10 forest classes; 20–25 traps per transect
Lubāna	2012–2016	11 habitats: 1 meadow and 10 forest classes; 20–25 traps per transect
Pļaviņas	2012; 2016	11 habitats: 1 meadow and 10 forest classes; 20–25 traps per transect
Augstroze	2012–2016	11 habitats: 1 meadow and 10 forest classes; 20–25 traps per transect

**Table 2:** Description of the influence of the prey abundance index on the FNB of owl species.

Owl Species	Prey (Index)	β ± SE	Test Statistic	df *	*p*-Value	AICc	R^2^_adj._/R^2^_marg._ **	R^2^_cond._	ICC
STRALU	Small mammals	−0.6127 ± 0.1850	−3.312	144.778	0.0012	662.308	0.063	0.068	0.005
	Microtus voles	−0.3886 ± 0.1551	−2.506	156.303	0.0132	667.001	0.037	0.049	0.013
	Bank voles	−0.2268 ± 0.0812	−2.795	161.449	0.0058	666.913	0.046	0.052	0.007
STRURA	Small mammals	−0.0039 ± 0.2514	0.015	54	0.9880	177.536	−0.019		
	Microtus voles	−0.1304 ± 0.1820	−0.716	54	0.4770	177.007	−0.009		
	Bank voles	−0.0050 ± 0.0837	−0. 06	54	0.9520	177.532	−0.019		
GLAPAS	Small mammals	−0.4948 ± 0.7738	−0.639	5	0.5507	39.803	−0.109		
	Microtus voles	−0.5290 ± 0.5286	−1.001	5	0.3628	39.075	0.0003		
	Bank voles	−0.4110 ± 0.2885	−1.425	5	0.2136	37.967	0.147		
ASIOTU	Small mammals	0.0171 ± 0.1531	0.112	22	0.9120	56.975	−0.045		
	Microtus voles	−0.0672 ± 0.1497	−0.449	22	0.6580	56.769	−0.036		
	Bank voles	0.0236 ± 0.0752	0.314	22	0.7570	56.881	−0.041		

* Satterthwaite’s degrees of freedom in LMM. ** R^2^_adjusted_ reported in the case of LM; R^2^_marginal_ in the case of LMM.

**Table 3:** Description of prey weight proportion in owl diet relative to the abundance index in nature (first two rows per owl species) or their cross correlation.

Owl Species	Prey (Index) *	β ± SE	Test Statistic	*p*-Value	AICc	R^2^_MF_/R^2^_marg._ **	R^2^_cond._	ICC
STRALU	Bank voles	0.0248 ± 0.0057	4.359	<0.0001	5693.822	0.0005	0.134	0.133
	Microtus voles	0.1302 ± 0.0061	21.520	<0.0001	14934.823	0.004	0.191	0.189
	Bank~Microtus	−0.0138 ± 0.0116	−1.189	0.2340	5711.112	<0.0001	0.134	0.134
STRURA	Bank voles	−0.1116 ± 0.0072	−15.570	<0.0001	4693.187	0.0581		
	Microtus voles	0.1275 ± 0.0100	12.720	<0.0001	6132.077	0.0268		
	Bank~Microtus	−0.1658 ± 0.0175	−9.497	<0.0001	4865.001	0.0208		
GLAPAS	Bank voles	−0.4168 ± 0.0717	−5.812	<0.0001	261.048	0.178		
	Microtus voles	0.2136 ± 0.0556	3.839	0.0001	220.591	0.073		
	Bank~Microtus	−0.9097 ± 0.0811	−11.220	<0.0001	145.446	0.613		
ASIOTU	Bank voles	−0.5294 ± 0.0497	−10.640	<0.0001	761.318	0.215		
	Microtus voles	0.0611 ± 0.0138	4.419	<0.0001	599.700	0.044		
	Bank~Microtus	−1.2718 ± 0.1208	−10.530	<0.0001	705.794	0.276		

* Prey type Bank~Microtus represents the proportion of bank voles in the diet depending on *Microtus* sp. vole abundance in nature. ** R^2^_McFadden_ reported in the case of GLM; R^2^_marginal_ reported in the case of GLMM.

**Table 4:** Description of owl population change trends with small mammal cycles (“before”) and since they vanished (“after”). Model coefficients are in log-odds scale.

Owl Species	Parameter	β ± SE	Test Statistic	*p*-Value	df *	R^2^_adj._ **
STRALU	Intercept	0.1178 ± 0.0396	2.978	0.0062	26	−0.1096
	Time	0.0016 ± 0.0043	0.368	0.7159		
	Before		reference			
	After	<0.0001 ± 0.0059	<0.0001	1		
	Time:After	<0.0001 ± 0.0006	<0.0001	1		
STRURA	Intercept	0.1389 ± 0.1361	1.020	0.319	21	0.5837
	Time	0.0319 ± 0.0179	1.783	0.089		
	Before		reference			
	After	1.1350 ± 0.1925	5.898	<0.0001		
	Time:After	−0.1354 ± 0.0253	−5.361	<0.0001		
GLAPAS ***	Intercept	0.0069 ± 0.07470	0.093	0.9279	11	0.4368
	Time	−0.0339 ± 0.0106	−3.210	0.0083		
ASIOTU	Intercept	−0.8450 ± 0.2688	−3.144	0.0046	23	−0.0302
	Time	−0.0143 ± 0.0328	−0.436	0.6666		
	Before		reference			
	After	0.3087 ± 0.3801	0.812	0.4250		
	Time:After	−0.0080 ± 0.0464	−0.172	0.8651		
AEGFUN	Intercept	−0.5246 ± 0.0331	−15.855	<0.0001	23	0.9740
	Time	−0.0607 ± 0.0040	−15.033	<0.0001		
	Before		reference			
	After	−0.7508 ± 0.0468	−16.043	<0.0001		
	Time:After	0.0183 ± 0.0057	3.211	0.0039		

* df is the same in each variable. ** Values represent the whole model. *** GLAPAS had data only since 2004.

**Table 5:** Description of owl breeding performance trends with small mammal cycles (“before”) and since their depletion (“after”).

Owl Species	Parameter	β ± SE	Test Statistic	*p*-Value	df *	R^2^_adj._ **
STRALU	Intercept	2.6221 ± 0.1594	16.452	<0.0001	23	0.0765
	Time	0.0174 ± 0.0194	0.896	0.380		
	Before		reference			
	After	−0.3597 ± 0.2254	−1.596	0.124		
	Time:After	0.0216 ± 0.0275	0.784	0.441		
STRURA	Intercept	1.8291 ± 0.1709	10.705	<0.0001	21	−0.0812
	Time	0.0106 ± 0.0224	0.473	0.641		
	Before		reference			
	After	−0.2022 ± 0.2416	−0.837	0.412		
	Time:After	0.0293 ± 0.0317	0.923	0.366		

* df is the same in each variable. ** Values represent the whole model.

**Table 6:** Spearman’s correlation analysis results of the annual mean number of fledglings and small mammal abundance indices in the year of breeding and the previous autumn (annotated as ^−1^).

Owl Species	Prey (Index)	r_s_	*p*-Value	Number of Years	S
STRALU	Small mammals	−0.1152	0.5737	26	3262
	Microtus voles	−0.1391	0.4962	26	3332
	Bank voles	0.0338	0.8700	26	2826
	Small mammals^−1^	0.1300	0.5341	25	2262
	Microtus voles^−1^	0.1377	0.5100	25	2242
	Bank voles^−1^	0.1946	0.3496	25	2094
STRURA	Small mammals	−0.0179	0.9340	24	2341.1
	Microtus voles	0.0545	0.8005	24	2174.8
	Bank voles	−0.0863	0.6886	24	2498.4
	Small mammals^−1^	−0.1788	0.4145	23	2385.8
	Microtus voles^−1^	−0.2104	0.3351	23	2449.9
	Bank voles^−1^	0.0218	0.9214	23	1979.9
ASIOTU	Small mammals	0.0805	0.7755	15	514.92
	Microtus voles	0.1252	0.6566	15	489.87
	Bank voles	0.0787	0.7804	15	515.92
	Small mammals^−1^	0.3062	0.2871	14	315.69
	Microtus voles^−1^	0.0529	0.8576	14	430.95
	Bank voles^−1^	−0.2643	0.3612	14	575.26
BUBBUB	Small mammals	0.5329	0.0408	15	261.59
	Microtus voles	0.3817	0.1604	15	346.28
	Bank voles	0.3402	0.2146	15	369.49
	Small mammals^−1^	0.6438	0.0130	14	162.09
	Microtus voles^−1^	0.5527	0.0404	14	203.50
	Bank voles^−1^	0.2020	0.4886	14	363.09

^−1^ Abundance index in the previous autumn.

## Data Availability

The aggregated (per species, per year) data presented in this study are available in the Supplementary Materials of this article. The raw data are not publicly available as we are not allowed to share raw monitoring information or nest locations.

## Supplementary Materials

The following supporting information can be downloaded at: https://www.mdpi.com/article/10.3390/life13020572/s1, Table S1: Description of owl diet per year; Table S2: Population indices of small mammals; Table S3: Population indices of owls; Table S4: Description of owl breeding performance.

## Appendix A

**Table A1:** Minimum legal rotation ages per site quality class in main tree species. Currently, there is no minimum rotation age in grey alder. We used 35 years, as it is the age of the youngest stand registered as “full grown” in FSR.

Dominant Tree Species	Highest Quality	Medium Quality	Lowest Quality
Oaks	101	121	121
Pines and larches	101	101	121
Spruces, ashes, limes, elms, and maples	81	81	81
Birches	71	71	51
Black alders	71	71	71
Aspens	41	41	41

**Table A2:** Marginal mean comparison (tests were conducted on the log scale; p adjusted by Tukey’s method) of the number of small mammals per 100 trap days between sample areas. The model with a random intercept per transect and main effects of sample area and year was considered the best *.

Sample Areas Contrasted	Ratio ± SE	z-Ratio	*p*-Value
Apsalas/Augstroze	0.704 ± 0.459	−0.538	0.9983
Apsalas/Kaunata	0.468 ± 0.307	−1.157	0.9099
Apsalas/Ķemeri	1.816 ± 1.587	0.682	0.9936
Apsalas/Lubāna	0.538 ± 0.35	−0.951	0.964
Apsalas/Pļaviņas	0.566 ± 0.371	−0.868	0.9772
Apsalas/Žūklis	1.329 ± 1.176	0.322	0.9999
Augstroze/Kaunata	0.665 ± 0.236	−1.148	0.9132
Augstroze/Ķemeri	2.578 ± 1.752	1.393	0.8057
Augstroze/Lubāna	0.764 ± 0.265	−0.774	0.9874
Augstroze/Pļaviņas	0.804 ± 0.286	−0.614	0.9964
Augstroze/Žūklis	1.887 ± 1.308	0.917	0.9701
Kaunata/Ķemeri	3.877 ± 2.65	1.983	0.4257
Kaunata/Lubāna	1.149 ± 0.408	0.393	0.9997
Kaunata/Pļaviņas	1.209 ± 0.439	0.522	0.9985
Kaunata/Žūklis	2.839 ± 1.978	1.498	0.7465
Ķemeri/Lubāna	0.296 ± 0.201	−1.79	0.5547
Ķemeri/Pļaviņas	0.312 ± 0.213	−1.705	0.6123
Ķemeri/Žūklis	0.732 ± 0.663	−0.344	0.9999
Lubāna/Pļaviņas	1.052 ± 0.373	0.142	1
Lubāna/Žūklis	2.47 ± 1.711	1.305	0.8498
Pļaviņas/Žūklis	2.349 ± 1.633	1.228	0.8834

* AICc = 1603.663, R^2^_conditional_ = 0.877, R^2^_marginal_ = 0.177, ICC = 0.851.

**Table A3:** Marginal mean comparison (tests were conducted on the log scale; p adjusted by Tukey’s method) of the number of small mammals per 100 trap days between forest age classes. The model with a random intercept per transect and main effects of forest age class and year was considered the best *.

Forest Age Groups Contrasted	Ratio ± SE	z-Ratio	*p*-Value
Young/Meadow	7.078 ± 2.772	4.997	<0.0001
Young/Medium	1.458 ± 0.442	1.244	0.599
Young/Old	1.627 ± 0.477	1.661	0.3446
Meadow/Medium	0.206 ± 0.073	−4.451	0.0001
Meadow/Old	0.23 ± 0.079	−4.257	0.0001
Medium/Old	1.116 ± 0.27	0.452	0.9692

* AICc = 1579.355, R^2^_conditional_ = 0.884, R^2^_marginal_ = 0.409, ICC = 0.804.

**Table A4:** Marginal mean comparison (tests were conducted on the log scale; p adjusted by Tukey’s method) of the number of small mammals per 100 trap days between soil richness classes. The model with a random intercept per transect and main effects of soil fertility class and year was considered the best *.

Soil Fertility Classes Contrasted	Ratio ± SE	z-Ratio	*p*-Value
Meadow/Fertile	0.153 ± 0.047	−6.086	<0.0001
Meadow/Poor	0.309 ± 0.098	−3.717	0.0006
Fertile/Poor	2.026 ± 0.397	3.604	0.0009

* AICc = 1572.443, R^2^_conditional_ = 0.879, R^2^_marginal_ = 0.529, ICC = 0.743.
